# Effects of niacin and omega-3 fatty acids on HDL-apolipoprotein A-I exchange in subjects with metabolic syndrome

**DOI:** 10.1371/journal.pone.0296052

**Published:** 2024-02-26

**Authors:** Mark S. Borja, Bradley Hammerson, Chongren Tang, Litzy Juarez-Serrano, Olga V. Savinova, William S. Harris, Michael N. Oda, Gregory C. Shearer

**Affiliations:** 1 Children’s Hospital Oakland Research Institute, Oakland, California, United States of America; 2 Department of Chemistry and Biochemistry, California State University East Bay, Hayward, California, United States of America; 3 University of Washington School of Medicine, Seattle, Washington, United States of America; 4 Cardiovascular Research Center, Sanford Research, University of South Dakota, Sioux Falls, South Dakota, United States of America; 5 OmegaQuant, Sioux Falls, South Dakota, United States of America; Endocrinology and Metabolism Population Sciences Institute, Tehran University of Medical Sciences, ISLAMIC REPUBLIC OF IRAN

## Abstract

HDL-apolipoprotein A-I exchange (HAE) measures a functional property associated with HDL’s ability to mediate reverse cholesterol transport. HAE has been used to examine HDL function in case-control studies but not in studies of therapeutics that alter HDL particle composition. This study investigates whether niacin and omega-3 fatty acids induce measurable changes in HAE using a cohort of fifty-six subjects with metabolic syndrome (MetS) who were previously recruited to a double-blind trial where they were randomized to 16 weeks of treatment with dual placebo, extended-release niacin (ERN, 2g/day), prescription omega-3 ethyl esters (P-OM3, 4g/day), or the combination. HAE was assessed at the beginning and end of the study. Compared to placebo, ERN and P-OM3 alone significantly increased HAE by 15.1% [8.2, 22.0] (*P*<0.0001) and 11.1% [4.5, 17.7] (*P*<0.0005), respectively, while in combination they increased HAE by 10.0% [2.5, 15.8] (*P* = 0.005). When HAE was evaluated per unit mass of apoA-I ERN increased apoA-I specific exchange activity by 20% (2, 41 CI, *P* = 0.02) and P-OM3 by 28% (9.6, 48 CI, *P<*0.0006). However the combination had no statistically significant effect, 10% (-9, 31 CI, *P* = 0.39). With regard to P-OM3 therapy in particular, the HAE assay detected an increase in this property in the absence of a concomitant rise in HDL-C and apoA-I levels, suggesting that the assay can detect functional changes in HDL that occur in the absence of traditional biomarkers.

## Introduction

Plasma levels of high-density lipoprotein cholesterol (HDL-C) are strongly and inversely associated with cardiovascular disease [1]. However, circulating HDL-C levels alone do not reflect the atheroprotective properties of HDL, as evidenced by the failures of cholesterol ester transfer protein (CETP) inhibitors and niacin [2,3] to improve cardiovascular outcomes despite significantly raising HDL-C levels. Furthermore, mutations in scavenger receptor B1 (SR-B1) that are associated with reduced hepatic clearance are also associated with very high HDL-C levels and elevated CVD risk [4], while mutations such as apolipoprotein (apo)A-I^Milano^ and lecithin:cholesteryl acyltransferase (LCAT) deficiency result in very low HDL-C but are not associated with increased CVD risk [5,6]. These findings underscore the complexities of HDL’s role in atheroprotection, highlighting the need to investigate HDL function, in addition to the more traditional measures.

Apolipopotein A-I (apoA-I) is the primary protein component of HDL comprising about 70% of its total protein mass. Plasma levels of apoA-I may be a better representation of HDL function than HDL-C, as demonstrated in a recent meta-analysis, wherein increases in apoA-I levels were correlated with reduced risk of CVD events but HDL-C levels were not [7]. ApoA-I exchanges between lipid-free and lipid bound states [8]. This process, herein termed HDL-apoA-I exchange (HAE), reports the rate at which resident apoA-I comes into equilibrium with exogenous lipid-free apoA-I. Assays measuring HAE have been performed using fluorescence-based approaches as well as electron paramagnetic resonance spectroscopy(EPR) [9,10]. Lipid-free and lipid-bound (i.e., HDL-associated) apoA-I bear distinct structural conformations and the HAE assay monitors this change in apoA-I structure as exogenous apoA-I displaces native apoA-I on HDL [10]. The HAE assay is independently associated with both ABCA1-mediated and total cellular cholesterol efflux capacity [11]. Presumably, this is because the primary acceptor of cholesterol and phospholipid from ABCA1 are small, dense HDL and lipid-poor apoA-I, whereas spherical HDL is a poor substrate for ABCA1 [12,13]. HDL’s ability to exchange its endogenous apoA-I to serve as a substrate for HDL biogenesis greatly impacts its ability to mediate cholesterol transport. Indeed, when HDL particles are oxidized, HAE and cholesterol efflux capacity both significantly decrease [14]. HAE and cholesterol efflux capacity are also impaired in the setting of metabolic syndrome (MetS), which is a major risk factor for atherosclerotic CVD [15].

To date, HAE has been used to investigate the functionality of HDL in several case-control studies, including in the setting of acute coronary syndrome [10], MetS [15], and type I and type II diabetes mellitus [16,17]. More recently, low HAE was associated with higher incidence of major adverse cardiovascular events [9]. However, HAE has not been examined in the setting of therapeutic intervention. The aim of this study was to investigate the effects of extended-release niacin (ERN) and prescription omega-3 fatty acid (P-OM3) alone or in combination, on HAE. Niacin and omega-3 fatty acids were selected because Shearer, et al. [18] showed that these therapeutics alter the lipoprotein profile of patients and thus are good candidates for inducing changes in HAE.

## Materials and methods

### Study subjects

Subjects with MetS (n = 56) were recruited as described [18]. This is an ancillary study. Inclusion criteria were age 40–69 years, body mass index (BMI) 25 to 40 kg/m^2^, fasting TG > 140 mg/dL, HDL-C > 10 mg/dL, and the ratio of TG/HDL-C > 3.5. Exclusion criteria were presence of secondary causes of dyslipidemia or hyperglycemia (e.g., hepatic, renal, thyroid or other endocrine diseases), cardiovascular disease, diabetes mellitus; history of hypersensitivity to niacin, fish oils, history of gout, hepatitis, peptic ulcer; use of any dietary supplement containing more than 50 mg of niacin or 100 mg of OM3 fatty acids; use of any herbal preparations or weight-loss products; use of any lipid-lowering drugs other than statins; medically-required treatment with nitrates, calcium channel blockers, or adrenergic blocking agents, hemoglobin < 12 g/dL; LDL-C > 145 mg/dL, or known substance abuse. The study was registered at clinicaltrials.gov (NCT00286234). The study was approved by the institutional review board at the University of South Dakota. Written informed consent was obtained prior to the study from all participants. Samples were collected between 2007 and 2008 and kept in storage at -80 ˚C without thawing. HAE measurements in this study were performed in 2012, and sample storage time did not affect the measurement of HDL function.

### Study design

Full study design details are available in Shearer, et al. [18]. Briefly, the study was a randomized, double-blind, placebo-controlled clinical trial with a 2 x 2 factorial treatment design. Group size was determined from a pilot study [19] and was originally powered for a non-HDL outcome (i.e., the rate of appearance of non-esterified fatty acids in the plasma). The power estimates from the pilot study for lipid endpoints are reported in Shearer, et al. [18] and were >80%. After initially qualifying for the study, subjects began a 6 week, diet-stabilization, dual-placebo, run-in phase (single blind) in which noncompliant subjects (i.e., <80% compliant) were to be identified and excluded (but none were). After the run-in phase subjects were randomly assigned (using permuted blocks of four with stratification for gender) to 16 weeks of treatment to either dual placebo, ERN (Niaspan, Abbott Labs, 2 g/d), P-OM3 (Lovaza, GlaxoSmithKline Pharmaceuticals, 4 g/d), or the combination. To improve tolerance, ERN was titrated up over the first month by adding 500 mg to the daily dose each week, to achieve the final 2 g/d. All subjects (placebo and active) were additionally asked to take aspirin (81 mg) prior to dinner to reduce flushing. To improve blinding to ERN, the ERN placebos contained 50 mg rapid-release niacin, which is enough to induce a mild flushing response.

Lipid data collection took place at weeks 0 (baseline) and 16 (endpoint). Subjects were asked to fast for at least 8 h prior to each visit. Blood was collected and plasma was separated within 30 min. The samples were stored at -70˚C prior to experimentation. Compliance to treatment was assessed by pill count for both treatments and also by changes in the OM3 index [20] which is the red blood cell fatty acid weight percent of (eicosapentaenoic acid) EPA and docosahexaenoic acid (DHA). Four subjects from the original study [18] were excluded because they lacked apoA-I quantitation data. One subject (assigned to placebo treatment) had baseline triglyceride (TG) levels above 500 and was excluded when analyses included TG. All subjects were >80% compliant based on pill count.

### Lipid and lipoprotein measurements

Plasma lipids and lipoproteins were measured using the Vertical Auto Profile technique (VAP; Atherotech, Birmingham, AL).

### ApoA-I quantitation

ApoA-I levels in subjects were quantified as previously described in Savinova, et al. [21]. Briefly, lipoproteins were isolated from EDTA plasma by sequential ultracentrifugation in densities 1.006; 1.063; and 1.21 g/ml corresponding to VLDL, IDL/LDL, and HDL fractions, and stored frozen (−80°C) until analysis. Lipoprotein fractions (4.5 μg of protein) were subjected to gradient SDS-PAGE (4–20% Peptide gels, BioRad, Hercules, CA) and stained with Sypro Orange (Invitrogen, Grand Island, N.Y.). Gels were scanned using Typhoon scanner at 532/555 nm excitation/emission wave lengths and analyzed using Image Quant version 5.0. Intensities of all bands were measured as area under the curve with baseline adjusted manually. The absolute amount of protein in each band was calculated based on its fraction of total protein loaded on the gel (4.5 μg per lane). Linear responses to apoplipoproteins were verified, r^2^ = 0.98. Protein identification was aided by LC-MS/MS, MALDI-TOF, and comparative 2D electrophoresis. By this method, six classic apolipoproteins: apoA-I, A-II, B, C-II, C-III, and E were consistently identified. The majority of apoA-I was detected in HDL and a minor amount was detected in the density fraction corresponding to LDL. Total apoA-I levels in plasma were calculated from these measurements.

### HDL-apoA-I exchange measurement

HAE was measured using EPR to quantify the binding of exogenous, spin-labeled apoA-I to HDL in plasma as described in Borja, et al. [10]. HAE is measured by directly quantifying the binding of exogenous, spin-labeled, lipid-free apoA-I to HDL, wherein there is a coincident (1:1) displacement of resident apoA-I from HDL particles [8,22]. ApoA-I binding to HDL is quantified by monitoring the center field peak intensity of the EPR spin-label’s spectra, whose intensity is conformation dependent. This signal increases linearly as exogenous apoA-I transitions from a lipid-free to lipid-bound conformation [10]. Briefly, freshly thawed plasma was mixed 1∶4 with PBS (20 mM phosphate, 150 mM NaCl, pH 7.4) and 24% w/v PEG 6000 (Sigma) was added to a final concentration of 4% and samples centrifuged at 4°C to remove apoB-containing lipoproteins. The clarified plasma was then mixed with 3 mg/mL spin-labeled apoA-I [23] in a 3:1 ratio and drawn into an EPR-compatible borosilicate capillary tube (VWR).

EPR measurements were performed with a Bruker eScan EPR spectrometer outfitted with temperature controller (Noxygen). Samples were scanned first at 6°C, incubated for 15 minutes at 37°C, and scanned again at 37°C. The baseline spectra of spin-labeled apoA-I in PBS was subtracted from results. Maximum amplitude of spin-labeled apoA-I was determined from spin-labeled apoA-I in a fully lipid-bound conformation. All samples were read in triplicate and averaged. HAE was calculated as described [10]. The inter-assay coefficient of variability was 6.4%.

### Statistical methods

Changes in response were modeled as dependent variables, with adjustment for baseline values to account for chance differences related to the small sample size per group. Because the power to detect interactions was low (<30%), the groups were tested using a one-way ANCOVA with Dunnett adjustment for multiple comparisons to the placebo group as in the parent study [18]. Residuals were examined for normality and homogeneity, and a natural log transformation was used as needed for improved model assumptions. *P*-values < 0.05 were considered statistically significant. Results are reported as least squares mean [95% CI] unless otherwise indicated. Statistical analyses were performed using JMP 8.0 (SAS Institute, Cary, NC) and GraphPad Prism 7.0 (GraphPad Software, San Diego, CA).

## Results

### Baseline characteristics

The baseline characteristics of the study subjects are summarized in Table 1. Fifty-six subjects were taken from the parent study [18], which included 60 subjects. Four subjects (one each from the placebo and ERN groups and two from the P-OM3 group) were excluded because we were unable to gather the relevant apoA-I data. Additional baseline data was previously reported in Shearer, et al. [18]. Age, statin and hypertensive drug use, HDL-C, and triglyceride (TG) levels were previously reported, but recalculated here to account for 4 fewer subjects. Notably, only 7 of the 56 subjects were on statins during the study. At baseline, mean values were not significantly different for any parameter among the treatment groups.

**Table 1:** Baseline characteristics by treatment group.

	Dual Placebo (n = 14)	ERN (n = 14)	P-OM3 (n = 15)	Combination (n = 13)
Age (years)	50 ± 13	47 ± 11	46 ± 11	49 ± 7
Sex: n (% male)	8 (57%)	8 (57%)	8 (53%)	9 (69%)
Statin use: n (%)	3 (21%)	2 (14%)	3 (20%)	0 (0%)
Anti-hypertensive use: n (%)	3 (21%)	4 (29%)	3 (20%)	3 (23%)
HDL-C (mg/dL)	41 ± 7	44 ± 10	44 ± 7	40 ± 8
Triglyceride (mg/dL)	254 ± 186	193 ± 66	206 ± 73	228 ± 82
ApoA-I (mg/dL)	93 ± 18	103 ± 21	101 ± 18	95 ± 18
% HAE	48.5 ± 12.4	53.8 ± 6.9	51.9 ± 9.2	43.8 ± 12.5

± indicates mean and SD; HDL-C indicates high-density lipoprotein cholesterol; apoA-I indicates apolipoprotein A-I; %HAE indicates the estimated percentage of spin-labeled apoA-I exchanging onto HDL; All *P* > 0.05.

### Effects of treatment on apoA-I levels

**Fig 1:**
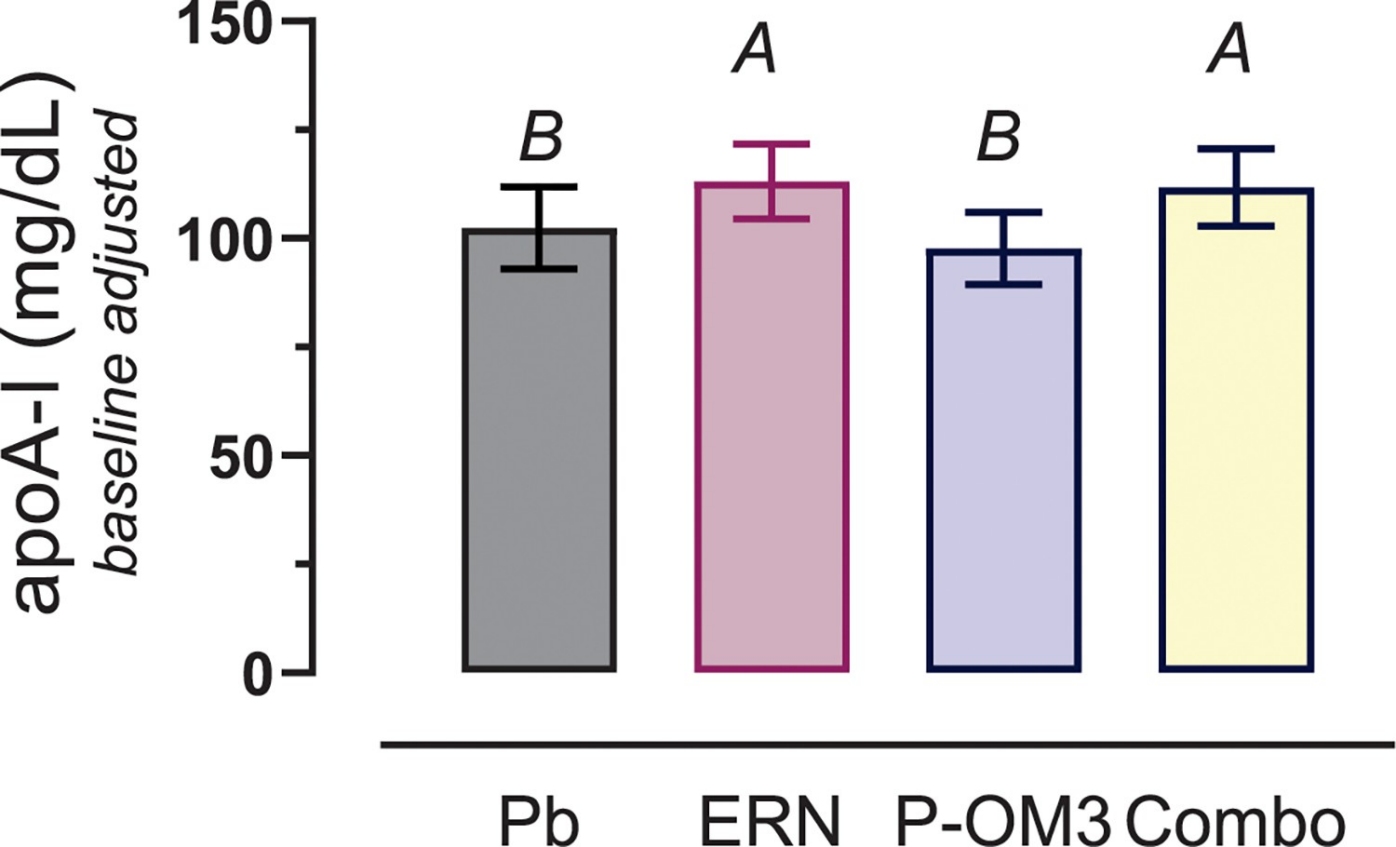
Change in apoA-I levels following 16 weeks of therapy with placebo (Pb), ERN, P-OM3, or combination (Combo). Statistical significance was determined using ANOVA adjusted for baseline (*P* = 0.04). The group differences were evident by post-hoc testing. Combined, ERN treatment (ERN and Combo: *A*) had increased apoA-I by 12 mg/mL (3, 20) *P* = 0.009) compared to groups with no niacin (*B*).

ApoA-I levels following 16 weeks of treatment are shown in Fig 1. There was a significant treatment effect (*P* = 0.04). Post-hoc testing clarified that this treatment effect was observed in subjects taking niacin (ERN and combination), where apoA-I increased by an average of 12 [4, 21] mg/mL (*P* = 0.008) compared to those not taking niacin (Placebo and P-OM3). As previously reported [18], ERN and combination therapy significantly raised HDL-C, while P-OM3 did not; and TG levels decreased significantly in all treatment groups, with combination therapy having the largest effect.

In an earlier study we showed that TG levels affect CEC measurement but not HAE.19 However, because of the significant reductions in TG levels in all treatment groups,22 we examined whether changes in HAE were due to decreases in TG. Normalizing for TG using a ratio of %HAE/TG, we found that treatment effects remained significant (S1 Fig) for ERN, P-OM3, and Combo treatment (P < 0.0001).

### Effects of treatment on HAE

**Fig 2:**
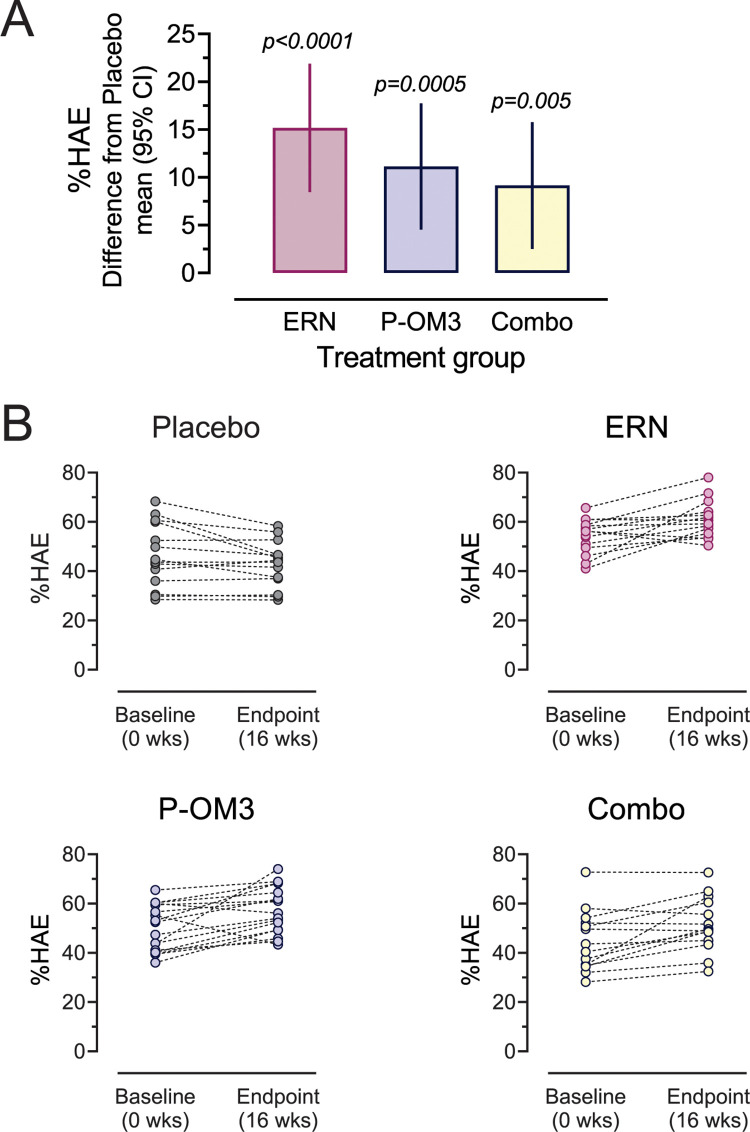
Effect of ERN, P-OM3, or combination therapy on HAE. **A**, the increase in %HAE relative to placebo adjusted for baseline %HAE, age, BMI, and sex. All treatments significantly increased %HAE (Dunnett test). **B**, the individual changes in HAE by treatment group.

All three treatment groups exhibited significant increases in HAE (Fig 2A). Plots showing the change in HAE for each individual are shown in Fig 2B. ERN increased HAE by 15.1% [8.5, 21.9 CI] (p<0.0001), while P-OM3 increased HAE by 11.1% [4.5, 17.7 CI] (p = 0.0005). Interestingly, the combination therapy group had a slightly smaller, but still significant increase in HAE at 9.1% [2.5, 15.8 CI] (*P* = 0.005).

In an earlier study we showed that TG levels affect CEC measurement but not HAE [15]. However, because of the significant reductions in TG levels in all treatment groups [18], we examined whether changes in HAE were due to decreases in TG. Adjusting for TG at baseline, we found that treatment effects remained significant, independent of TG levels for ERN and P-OM3 monotherapies (*P* < 0.0001), with slight attenuation for the combination therapy (*P* = 0.005).

### P-OM3 treatment independently increases HAE per unit mass of apoA-I

**Fig 3:**
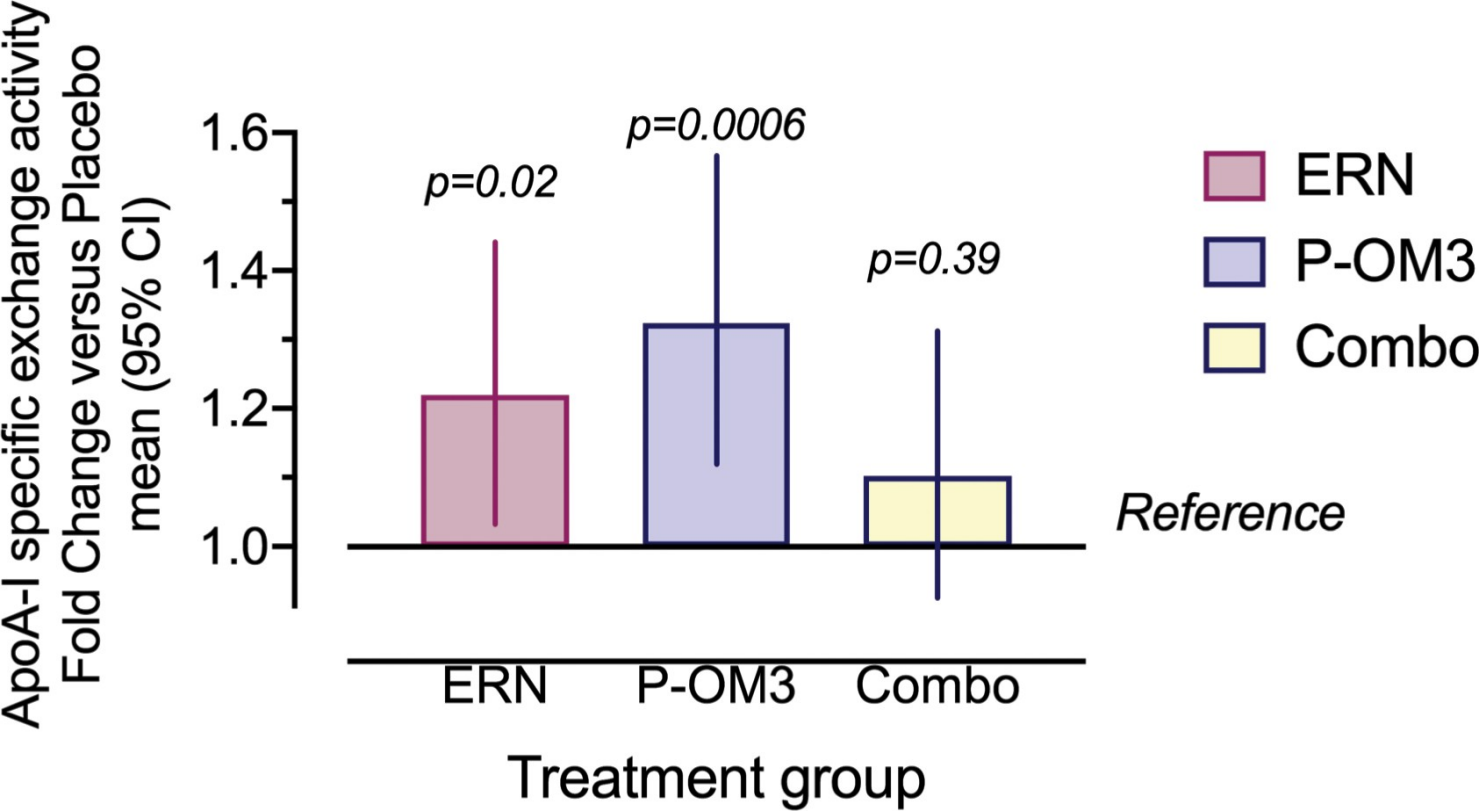
Effect ERN, P-OM3, and combination therapies on apoA-I specific exchange activity. ApoA-I specific exchange activity is calculated as %HAE / mg/dL apoA-I and shown as fold change from placebo. ERN therapy was 20% higher after treatment (P = 0.02), while P-OM3 exhibited a 28% increase in apoA-I specific activity (P = 0.0006). Combination therapy increased non-significantly (10%).

Because HAE is a measure of total exchange of apoA-I on and off HDL in a sample, subjects with high levels of apoA-I but with a low exchange rate will present with HAE similar to subjects with lower levels of high exchange rate apoA-I. It is therefore helpful to measure HAE in terms of apoA-I specific exchange activity, which is done by dividing the HAE value by the unit mass of apoA-I. This metric can be used to indicate whether an increase in HAE is due to increased apoA-I levels or increased activity per unit apoA-I [16], and provides a highly distinguishing measure of HDL functionality between healthy subjects and those at risk for CVD. Baseline apoA-I specific exchange activity values are reported in Table 1. Following treatment, apoA-I specific exchange activity in the native plasma exhibited a strong treatment-related effect (Fig 3, *P* = 0.005). P-OM3 exhibited the largest increase in apoA-I specific exchange activity at 28% (9.6,48 CI, *P* = 0.0006). ERN treatment increased apoA-I specific exchange activity by 20% (2,41 CI, *P* = 0.02). While combination therapy raised specific exchange activity by 10%, the effect was not significant (-9, 31 CI, *P* = 0.39). Furthermore, apoA-I specific exchange activity was significantly higher for P-OM3 treatment compared to combination (*P* = 0.006).

## Discussion

This is the first study to investigate the impact of therapeutic interventions on HAE. We examined ERN and P-OM3 alone and in combination in subjects with MetS, a population previously shown to have lower HAE compared to healthy individuals [15], and increased risk of CVD [24]. We found that all three treatments increased HAE. This remained significant even when the data were adjusted for baseline apoA-I and treatment-dependent change in apoA-I levels. Increases in HAE also remained significant when the data were adjusted for baseline and treatment-dependent change in TG. While combination therapy showed increased HAE compared to baseline, its positive effect was less than that of ERN and P-OM3 monotherapies.

HAE is strongly correlated with apoA-I levels [11]. and in this study, we observed differential effects of treatment on apoA-I levels. Treatment groups that included niacin (ERN and combination) saw significant increase in apoA-I levels following treatment. The ERN-specific effect of raising apoA-I has been observed previously with fenofibrate-ERN combination therapy but not with fenofibrate monotherapy [25]. P-OM3 did not raise apoA-I levels but increased HAE, demonstrating that significant changes in HAE are not necessarily coupled to significant changes in apoA-I levels.

ApoA-I is an important driver of RCT [26]. Lipid-poor apoA-I is the preferred substrate of ABCA1 [12], and apoA-I is also important for the remodeling and maturation of larger HDL subspecies [27]. Taking apoA-I levels into account when measuring HAE allows the calculation of apoA-I specific exchange activity, that is, the amount of exchange with respect to the concentration of apoA-I (measured in mg/dL in this study) in a given sample. This is a potentially important measure with regard to HDL function and CVD risk, as we have shown in earlier studies that apoA-I specific exchange activity is reduced in patients with acute coronary syndrome (ACS) [10], metabolic syndrome [15], type I diabetes mellitus [16], type II diabetes mellitus [17], and when apoA-I is enzymatically oxidized [8]. ApoA-I specific exchange activity may therefore provide a measure of a drug’s actual efficacy with respect to apoA-I exchange and its relevance to HDL’s ability to mediate RCT.

ERN and P-OM3 treatment enhanced HAE to a similar degree when HAE data were examined alone. However, when apoA-I specific exchange activity was examined, P-OM3 had the strongest effect, with a 28% increase over baseline. This is notable, as it represents a rise in HDL function that is independent of a concomitant increase in HDL-C or apoA-I levels. ERN significantly improved apoA-I specific exchange activity to a slightly lesser extent (20%).

The results with combination therapy were perplexing, having the smallest increase in HAE of the three treatment groups at 9.1%. ApoA-I specific exchange activity increased by 10% over baseline, but did not rise to the level of significance. In the previous study, combination therapy raised HDL-C and lowered TG to a greater extent than either of the monotherapies [18]. A potential explanation of the results with combination therapy may be due to the specific mechanisms by which ERN and P-OM3 alter HDL function. In addition to increasing HDL-C, niacin increases HDL particle size, converting small particles into larger HDL particles [28]. OM3 has the opposite effect, decreasing HDL particle size [29]. Additionally, OM3 increases the fluidity of the lipid bilayer in HDL [30], and bilayer fluidity is associated with increased cholesterol efflux [31]. Along these lines, reconstituted HDL particles incorporating eicosapentaenoic acid were reported to increase cholesterol efflux [32]. Cholesterol efflux capacity and HAE are strongly correlated [11], suggesting that the presence of OM3 in the HDL particle may explain the increase the rate of HAE. While future studies are necessary, these findings suggest that the lipid content of HDL particles may play a significant role in improving HDL functions associated with RCT.

Our findings may provide a mechanistic explanation for the differential results reported in clinical trials of niacin and OM3 therapeutics over the years. Clinical trials of niacin such as AIM-HIGH and HPS2-THRIVE found that while ERN significantly raises HDL-C and lowers TG, it does not reduce residual CVD risk [3,33]. On the other hand, P-OM3 therapy, icosapent ethyl in particular, showed reduction of residual risk in the absence of significant effects on traditional biomarkers except for TG [34,35]. Both ERN and P-OM3 raise HAE per mg/dL of apoA-I, but P-OM3 does so to a greater extent, which potentially explains findings that P-OM3 is associated with regression of fibrous plaques in combination with low-dose statins [36]. The HAE assay is predominantly independent of traditional biomarkers of CVD risk (importantly TG [15]) and may therefore be a good indicator of CVD residual risk, as the increase in apoA-I specific activity was greatest for the treatment that has shown the greatest reduction in residual risk in other studies (P-OM3 alone).

Strengths of this study include the careful selection of MetS subjects, the randomized study design and utilization of an assay permitting quantification of apoA-I specific exchange activity. The primary limitation of this study was the relatively small sample size, which reduced the power to detect interactions, that is, effects of the combination therapy beyond those that are simply additive. An additional limitation is that our study used whole omega-3 fatty acids, whereas only EPA (icosapent ethyl) has shown clinical benefit [37]. However, the aim of our study was not to demonstrate clinical benefit, but simply to determine whether changes in HAE could be observed following therapeutic intervention.

In conclusion, the HAE assay identified changes in HDL function associated with ERN and P-OM3 therapy. Both ERN and P-OM3 therapies increase HAE, alone and in combination. ApoA-I specific exchange activity was significantly increased with ERN and P-OM3 monotherapies, with the latter having the greatest increase, occurring in the absence of a concomitant rise in HDL-C or apoA-I levels. These findings suggest that it may be possible to improve HDL function in the absence of quantitative increases in HDL-C and apoA-I, and that measuring HDL function may play an important role in discovering therapies that do so.

## Supporting information

S1 Fig Effect of ERN, P-OM3, and combination therapies on the HAE/TG ratio following baseline adjustment. Statistical significance was determined using ANOVA following adjustment for baseline %HAE/TG (P **=** 0.04). %HAE/TG was log-transformed for normality and homoscedasticity. The group differences were evident by post-hoc testing. Compared to placebo, ERN treatment increased %HAE/TG 1.86-fold (P<0.001); P-OM3 increased %HAE/TG 1.70-fold (P<0.0001); and Combo increased %HAE/TG 2.02-fold (P **=** 0.0001).
(TIF)
