# Supplementary figures and images for: Effects of niacin and omega-3 fatty acids on HDL-apolipoprotein A-I exchange in subjects with metabolic syndrome
Source: PLoS One. 2024 Feb 26;19(2):e0296052. doi: 10.1371/journal.pone.0296052 (PMC10896500; doi:10.1371/journal.pone.0296052)

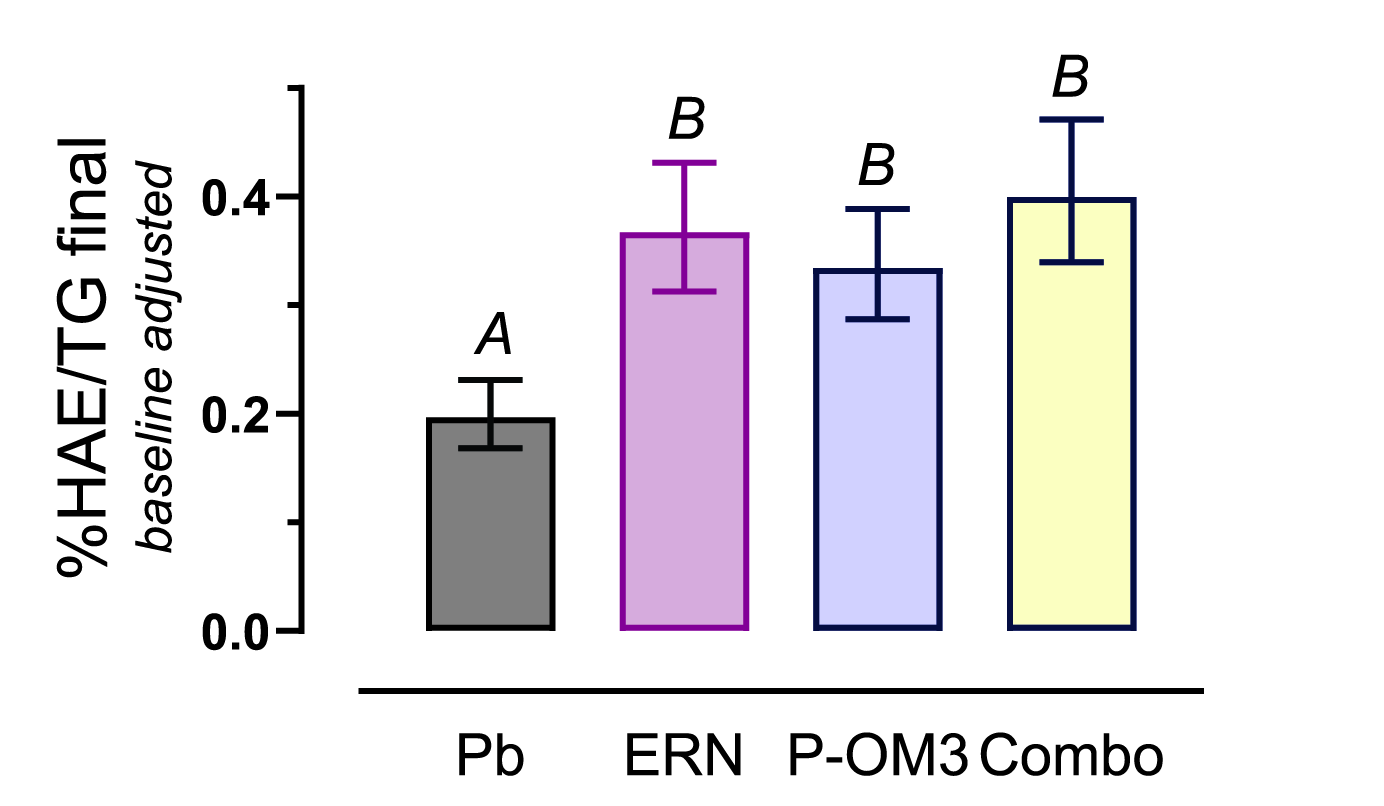

Supplement: S1 Fig — Statistical significance was determined using ANOVA following adjustment for baseline %HAE/TG (P = 0.04). %HAE/TG was log-transformed for normality and homoscedasticity. The group differences were evident by post-hoc testing. Compared to placebo, ERN treatment increased %HAE/TG 1.86-fold (P<0.001); P-OM3 increased %HAE/TG 1.70-fold (P<0.0001); and Combo increased %HAE/TG 2.02-fold (P = 0.0001). (TIF) [file pone.0296052.s001.tif]
